# Case report: ISL2 is involved in malignant transformation in a patient with multiple relapsed oligodendroglioma

**DOI:** 10.3389/fonc.2022.969191

**Published:** 2022-07-28

**Authors:** Shu-Na Chen, Zhongyong Wang, Di-Sheng Zhou, Xue-Qi Liu, Tao-Yi Mai, Zhao-Xia Dong, Miao Li, Xing-Ding Zhang, Lin Qi

**Affiliations:** ^1^ Department of Pharmacology, Molecular Cancer Research Center, School of Medicine, Sun Yat-sen University, Shenzhen, China; ^2^ Department of Neurosurgery, the Second Affiliated Hospital of Soochow University, Suzhou, China; ^3^ The Eighth Affiliated Hospital, Sun Yat-sen University, Shenzhen, China; ^4^ Department of Hematology, The First Affiliated Hospital of Shenzhen University, Shenzhen Second People’s Hospital, Shenzhen, China

**Keywords:** ISL2, angiogenesis, malignant transformation, oligodendroglioma, case report

## Abstract

The majority of oligodendrogliomas exhibit an intrinsic tendency to develop into malignant high-grade tumors. Angiogenesis is a major factor contributing to the malignant transformation of oligodendroglioma, and its molecular regulatory mechanism needs further study. We provide a case report of an oligodendroglioma patient with two recurrences whose disease progressed from WHO grade II to grade III. We showed that the expression of insulin gene enhancer protein (ISL2) and its angiogenic ability were positively correlated with the progression of oligodendroglioma. In Low-grade glioma (LGG) patients, including oligodendroglioma patients, overexpression of ISL2 was correlated with poor prognosis, and this correlation was not affected by gender or isocitrate dehydrogenase 1(IDH1) mutation status. ISL2 expression and ISL2-mediated angiogenic pathway activity are ideal biomarkers for the malignant transformation of oligodendroglioma. Anti-ISL2 therapy is also a potential treatment option for malignantly transformed oligodendroglioma.

## Introduction

Low-grade gliomas (LGGs) are slow-growing grade II or III primary brain tumors that mainly include astrocytoma, oligodendroglioma and oligoastrocytoma (1, 2). The key characteristic of oligodendrogliomas is mutation of isocitrate dehydrogenase (IDH) 1/2 and codeletion of chromosome arms 1p and 19q (3). LGGs generally undergo malignant transformation to high-grade invasive gliomas (grade III or grade IV), such as glioblastoma (GBM) (4). Approximately 60% of LGG patients with IDH mutations treated with temozolomide acquire a hypermutated genotype, and the increased mutational burden is associated with increased malignancy (5). Therefore, it is necessary to investigate the mechanism underlying the malignant transformation of oligodendroglioma.

ISL2, an islet-class LIM homeodomain transcription factor, has essential roles in motor neuron diversification and neuronal specification in the embryonic spinal cord (6, 7). It has been reported that the expression of ISL2, Tbr2, or Satb1/Satb2 determines the type of retinal ganglion cell by dividing them into three distinct functional classes (8). ISL2 affects the direction selectivity and innervation of retinal ganglion cells (9). The angiogenesis, proliferation, and invasion of human brain microvessel endothelial cells are mediated by VEGFA-ERK signaling, which is transcriptionally regulated by ISL2 (10).

Angiogenesis, the formation of new blood vessels from preexisting vessels, involves the proliferation of endothelial cells and vessel growth (11). Myeloid-derived VEGF-A plays an important role in the formation of abnormal vascular networks and the promotion of glioma growth (12). Bevacizumab, a humanized monoclonal antibody targeting human VEGF, fails to improve the survival of glioma patients as monotherapy but provides a benefit when combined with chemotherapy and immunotherapy (13). In our previous study, ISL2 was found to induce the transcription of ANGPT2 to promote the malignant transformation of oligodendroglioma through angiogenesis and proliferation; these findings provided mechanistic insights into antiangiogenic therapeutic strategies for gliomas (14).

To further verify the important role of ISL2-mediated angiogenesis in the malignant transformation of oligodendrogliomas, we report a patient with recurrent oligodendroglioma whose glioma progressed from WHO grade II to grade III. Specifically, we evaluated the correlations of ISL2 expression and ISL2-mediated angiogenic pathway activity with the malignant transformation of oligodendroglioma. Our results are of great significance for the detection of malignant transformation in oligodendroglioma and its treatment.

## Case presentation

### Patient information

**Figure 1:**
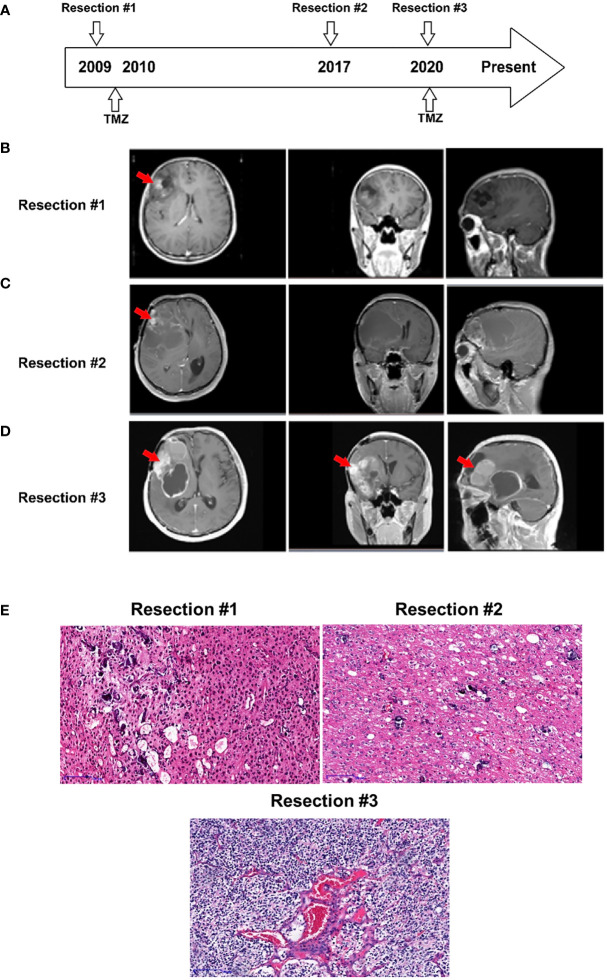
Case presentation. **(A)** Timeline of disease diagnosis and treatment. **(B–D)** Magnetic resonance imaging (MRI) obtained during three resections. The red arrows indicate the tumor locations. **(E)** Slides of resected tissue collected from the same patient at three timepoints in the disease course were subjected to HE staining (100X).

A 39-year-old man, presented with progressive decline in binocular vision at 24-year-old, was first diagnosed with oligodendroglioma, WHO grade II, at 27 years of age in 2008. As shown in **Figure 1A**, he underwent the initial tumor resection of the right frontal lobe. Magnetic resonance imaging (MRI) revealed a heterogeneous tumor mass measuring 4 x 4 x 4 cm in the right lobe (**Figure 1B**). The tumor tissue was grayish white, soft in texture, and rich in blood supply, and the boundary with normal brain tissue was diffuse. The tumor showed positivity for GFAP, Vin, S-100, EGFR, EMA and CD34 expression and was negative for CKpan and AE1/AE3 expression. The patient received temozolomide and radiation therapy (RT) for six months after resection.

After 8 years, in 2016, the patient experienced disease progression in the frontotemporal lobe region above the sphenoid ridge and the front-end of the frontalis muscle. The MRI scan further confirmed that the tumor recurred and was significantly larger (**Figure 1C**). The patient underwent a second gross total resection and was diagnosed with recurrent astrocytoma with calcification, WHO grade II. The tumor had similar characteristics to the first one except for the grayish-red color.

Two years prior to the current presentation, in 2020, the MRI scan showed an enhancing lesion in the right frontal lobe that was significantly increased in size, suggesting a worsening high-grade glioma (**Figure 1D**). The new mass, measuring 7 x 6 x 6 cm in the tumor bed, was resected, and a 30 ml intracerebral hematoma was cleared. The resected tumor, with the same characteristics as the previous tumor, was grayish yellow. The patient was diagnosed with recurrent oligodendroglioma with stroke, WHO grade III. The pathologic exam showed that the tumor underwent malignant transformation from an early disease stage (1^st^ and 2^nd^ resected tumors) to a late disease stage (3^rd^ resected tumor) (**Figure 1E**) and was positive for GFAP, Ki-67, S-100, Syn and Olig-2 expression and negative for CD99, EMA, CgA, AE1/AE3 and LCA expression.

### ISL2-mediated angiogenic pathway activity correlates with the malignant transformation of oligodendroglioma

**Figure 2:**
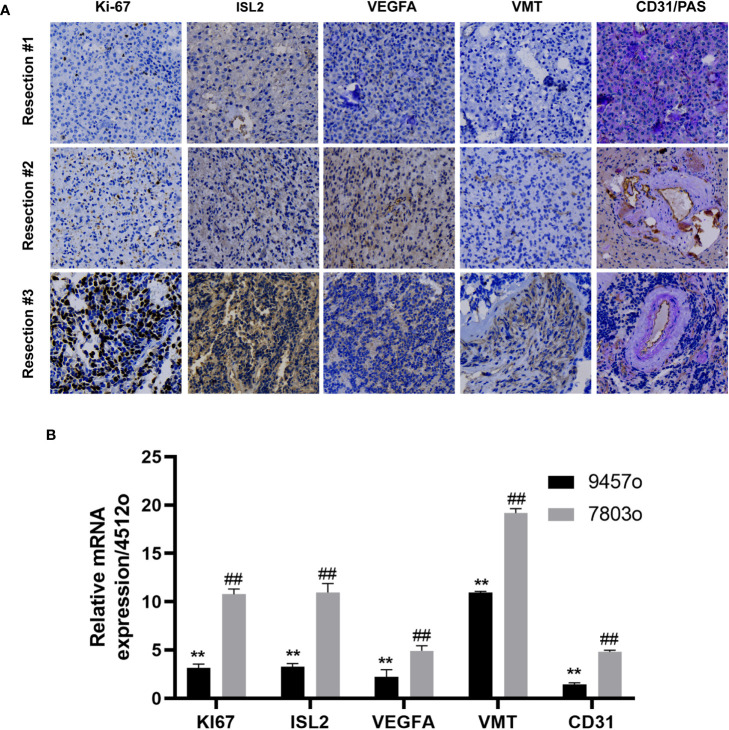
ISL2 was highly expressed with recurrence of oligodendroglioma. **(A)** Histopathological examination. The expression of Ki-67, ISL2, VEGFA, VMT and CD31/PAS in specimens collected from the same patient at three timepoints in the disease course was evaluated by IHC staining (40X). **(B)** The mRNA expression of Ki-67, ISL2, VEGFA, VMT and CD31 in specimens collected from the same patient at three timepoints in the disease course was determined by RT–PCR. ^**^p<0.002; ^##^p<0.002.

In a previous study, we reported that ISL2 regulates angiogenesis to promote the malignant transformation of oligodendroglioma (14). To confirm whether ISL2 regulates tumor progression through angiogenesis, we collected specimens from this patient at different timepoints in the disease course. We evaluated the expression of ISL2, VMT and the cell proliferation marker Ki-67. In addition, staining for VEGFA, CD31 and PAS, the markers of angiogenesis and vascular mimicry (VM), was performed (15, 16). The results showed that the expression of Ki-67 gradually increased with increasing disease duration. ISL2 was mainly expressed in specimens with high Ki-67 expression, which confirmed that ISL2 was more highly expressed in patients with malignantly transformed oligodendrogliomas. IHC staining of VEGFA and VMT revealed that angiogenesis was positively correlated with ISL2 expression and tumor progression. VM was identified by presentation as pink vascular-like structures with purple tumor cell linings. PAS and CD31 were highly expressed in patient specimens, indicating that VM and angiogenesis are common in oligodendroglioma. PAS/CD31 double staining indicated that VM has the ability to fuse with endothelium-forming blood vessels to form more abundant blood vessels in the more depraved tumors (**Figure 2A** and **Table 1**). The relationships of the expression levels of ISL2 and angiogenesis-related mRNAs with the malignant disease course was analyzed. The mRNA expression of ISL2 was significantly increased with increasing disease duration, accompanied by high expression of Ki-67, VEGFA, VMT and CD31 in the specimens, as determined by RT–PCR (**Figure 2B**). Collectively, these results showed that ISL2 and angiogenesis were associated with the malignant transformation of oligodendroglioma in this patient.

**Table 1:** Immunohistochemical findings.

Tumor	Ki-67	ISL2	VEGFA	VMT	CD31	PAS
Resection # 4512o	+(17%)	+(18%)	+(22%)	+(12%)	+(23%)	+(22%)
Resection # 9457o	+(18%)	+(52%)**	++(63%)*	+(17%)	++(32%)	++(43%)*
Resection # 7803o	+++(82%)***	+++(85%)***	++(72%)***	++(62%)**	+++(48%)*	+++(80%)***

Intensity of staining: week (+); moderate (++); strong (+++); Percentage represents the proportion of positive cells; 4512o, 9457o and 7803o represent the first to third surgical resection the tumors, respectively; n=3, *p<0.033; **p<0.002; ***p<0.001.

### ISL2 expression correlates with poor overall survival in LGG patients

**Figure 3:**
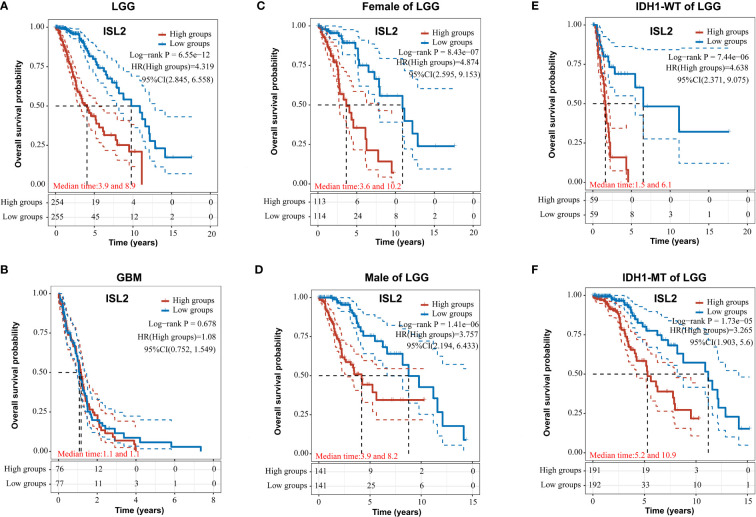
ISL2 correlates with poor overall survival in LGG patients. **(A, B)** The prognostic significance of ISL2 in LGG **(A)** and GBM **(B)** glioma tissues was analyzed in the TCGA database. (n (LGG)=509; n (GBM)=153). **(C, D)** The overall survival of female and male LGG patients with different ISL2 expression levels (n (female)=227; n (male)=282). **(E, F)** Kaplan–Meier survival curve showing the effects of isocitrate hydrogenase 1 (IDH1) status on overall survival in LGG patients with high versus low ISL2 expression levels. WT, wild-type; MT, mutated. HR (High exp) represents the risk coefficient of the samples in the high expression group relative to the samples in the low expression group: if HR > 1 means the gene is a risk factor, if HR < 1 means the gene is a protective factor. 95% CL represents the HR confidence interval; median time represents the time (in years) corresponding to a survival rate of 50% in both the high and low expression groups.

To evaluate the functions of ISL2 in gliomas, we investigated the overall survival (OS) of patients in a dataset from The Cancer Genome Atlas (TCGA) (https://portal.gdc.cancer.gov/). In LGG, the OS of patients with high ISL2 expression (ISL2^High^) was significantly worse than that of patients with low ISL2 expression (ISL2^Low^) (**Figure 3A**). In contrast, there was no difference in prognosis between the ISL2^High^ and ISL2^Low^ patient groups in GBM (**Figure 3B**). To study whether gender affects the negative correlation between ISL2 expression and the OS of patients, we analyzed the clinical information of female and male LGG patients separately. Kaplan–Meier survival analysis revealed that the median survival times of female and male LGG patients were similar (**Figures 3C, D**). In recent studies, it was found that the majority of LGGs harbored mutations in IDH1 and far fewer in IDH2 (17). Considering that the expression of ISL2 in IDH wild-type glioma was higher than that in IDH mutant glioma (10), we examined the OS of patients with IDH1 wild-type and mutant glioma. Kaplan–Meier analysis showed that IDH1 mutation status did not affect the negative correlation between ISL2 expression and OS (**Figures 3E, F**). The results suggested that the relationship between ISL2 expression and OS is independent of both gender and IDH1 mutation status.

## Discussion

In the present research, we reported that the expression levels of ISL2 and angiogenesis-related genes increased with the malignant transformation of oligodendroglioma. Furthermore, we showed that ISL2 expression was negatively correlated with the overall survival of oligodendroglioma patients. These results support the idea that ISL2 can be regarded as a biomarker for oligodendroglioma progression, suggesting that targeting ISL2 may provide a promising treatment option for malignantly transformed oligodendroglioma.

A variety of factors are known to contribute to the malignant transformation of oligodendrogliomas. Adjuvant therapy, especially adjuvant temozolomide monotherapy, is a risk factor for malignant transformation in LGG patients (18, 19). Inflammatory factors are also drivers of the malignant transformation of LGG by promoting migration, metastasis and angiogenesis (20). Similarly, angiogenesis mediated by CX3CR1 or ISL2 signaling plays a key role during the malignant transformation of LGG (10, 14, 21). Our study also verified the important roles of ISL2 and angiogenesis in oligodendroglioma progression.

Similar to indolent tumors, LGGs always develop into high-grade gliomas (HGGs) (17). It has been reported that the genetic changes in gliomas, particularly mutations in IDH, TP53, ATRX, CIC, and FUBP1, play a significant role in the progression from LGG to HGG (5, 22). Considering the impact of genomic heterogeneity on tumor progression, selecting clinical samples with a consistent genetic background is crucial for cancer research. However, the genetic background of clinical samples used in most research is complex and diverse, mainly due to the difficulty of collecting samples from the same patient with multiple relapses or from patients with the same genetic background. In our study, we collected three groups of slices from the same patient at different timepoint in the disease course. Our results identified the roles of ISL2 and angiogenesis in promoting the malignant transformation of oligodendroglioma independent of the influence of genetic background.

The oncogenic mechanisms of low-grade glioma are the failure of brain progenitor cells to develop into postmitotic neuroglial lineages and the proliferation of undifferentiated stem-like tumor cells, which results from the combination of IDH mutation and loss of P53 and ATRX (23). In contrast to astrocytomas, oligodendrogliomas with IDH mutation and 1p/19q codeletion exhibit less aggressive behavior, but patients do exhibit significant neurological deterioration and mortality (24, 25). In this study, we focused on the relationship between the role of ISL2 and the expression status of IDH1. Our results showed that the expression of ISL2 was negatively correlated with patient survival regardless of whether IDH1 was mutated. LGGs with wild-type IDH can be classified into two types. One type behaves almost identically to GBMs because it exhibits amplification of EGFR and K27M mutation of H3F3A, or TERT. In patients with this type, the average survival time is 1.23 years (26). Patients with the other type, without these mutations, have higher survival rates, and these tumors resemble LGGs in terms of their clinical course (24, 26). Moreover, ISL2 expression in GBMs was significantly higher than that in LGGs (10). Therefore, we speculated that the negative correlation between the ISL2 level and the overall survival of patients is mainly determined by the glioma status in patients with IDH wild-type LGG. In summary, the molecular regulatory mechanism of ISL2 in the malignant transformation of LGG is different from that of IDH1, a finding that requires further research.

## Conclusion

Our research indicated that ISL2 and angiogenesis were associated with the malignant transformation of oligodendroglioma. ISL2 was highly expressed in the more malignant LGGs and associated with poor patient survival. The molecular regulatory mechanism of ISL2 is unique, and is not influenced by patient gender or the status of IDH1. Our studies identify biomarkers for the detection of disease progression and therapeutic targets in oligodendroglioma.

## Data availability statement

The original contributions presented in the study are included in the article/**supplementary material**. Further inquiries can be directed to the corresponding author/s.

## Ethics statement

The studies involving human participants were reviewed and approved by the Ethics Review Board of Sun Yat-sen University. The patients/participants provided their written informed consent to participate in this study. Written informed consent was obtained from the individual(s) for the publication of any potentially identifiable images or data included in this article.

## Author contributions

SC and ZW performed the experiments and manuscript writing. DZ, XL and TM conducted the data analysis. ZD and ML revised the paper and provided some recommendations. LQ and XZ conceived and designed the study. All authors had read and approved the final manuscript. All authors contributed to the article and approved the submitted version.

## Funding

This work was supported in part by the National Natural Science Foundation of China (grant no. 32100563); the youth medical doctors project of Jiangsu province of China (grant no. QNRC2016870); and the project of Suzhou health talents of China (grant no.2020090, GSWS2021014).

## Conflict of interest

The authors declare that the research was conducted in the absence of any commercial or financial relationships that could be construed as a potential conflict of interest.

## Publisher’s note

All claims expressed in this article are solely those of the authors and do not necessarily represent those of their affiliated organizations, or those of the publisher, the editors and the reviewers. Any product that may be evaluated in this article, or claim that may be made by its manufacturer, is not guaranteed or endorsed by the publisher.
