# Supplementary material for: Case report: ISL2 is involved in malignant transformation in a patient with multiple relapsed oligodendroglioma
Source: Front Oncol. 2022 Jul 28;12:969191. doi: 10.3389/fonc.2022.969191 (PMC9366390; doi:10.3389/fonc.2022.969191)
Supplement: Supplementary file 1 [file DataSheet_1.docx]

Supplementary Material

# Supplementary Data

## Sample Resource

Three formalin fixation and paraffin embedding (FFPE) specimens from oligodendroglioma patients collected during 2008, 2016, and 2020 were selected for this study. The samples were removed during surgery, fixed with formalin for 24 hours and embedded in paraffin (Solarbio, Beijing, China). Paraffin blocks were sliced into serial sections. All sections were sterilized, and six FFPE sections of 5 µm in thickness were placed in block storage box tubes for backup. This protocol was approved by the Ethics Review Board of Sun Yat-sen University (Ethics Committee reference number 2020-SZMED-2020058) and run according to the standards of Good Clinical Practice as set out in the Declaration of Helsinki. Written consent for study participation was received from the patients or their guardians.

## Staining of tissue sections

H&E staining was performed according to the manufacturer's (Sigma/Aldrich) protocol. Immunohistochemical (IHC) staining was performed as described previously (1). The paraffin sections were dehydrated through an alcohol gradient and dewaxed with xylene. H_2_O_2_ (3 %) and a blocking agent were added and incubated at room temperature for 10 min. Subsequently, the slides were incubated with the following primary antibodies at 4 °C overnight：Ki67 (1:200) (CST, 9449s), ISL2 (1:300) (ThermoFisher, PA5-47599), CD31 (1:200) (Arigobio, ARG52748), VEGFA (1:200) (Santa Cruz, sc-7269) and Vimentin (VMT) (1:200) (CST, 5741). The appropriate biotinylated secondary antibodies were then applied and incubated for 30 min. The final IHC staining signals were detected using a 3,3’-diaminobenzidine (DAB) substrate kit (ABsin, abs957) according to the manufacturer's protocols. Finally, the sections were restained with hematoxylin and sealed with neutral gum. IHC staining was assessed by measuring the intensity and extent of immunopositivity.

Periodic acid-Schiff (PAS) staining was performed using a periodic acid-Schiff detection kit (Leagen, DG0005) according to the manufacturer’s protocol. The paraffin sections were immersed in periodic acid at room temperature for 5 min. After washing three times with distilled water, the slides were immersed in Schiff reagent for 20 min in the dark. Subsequently, the sections were restained with hematoxylin and sealed with neutral gum. PAS was assessed consistent with the above protocol for assessing IHC staining.

## Quantitative RT–PCR

Total RNAs were extracted using DNA/RNA FFPE Kit (Qiagen) following the manufacturer’s instructions and reverse transcribed into cDNA using cDNA Synthesis Kit (Thermo Fisher Scientific). Ki67 (GenBank accession no. AJ567756.1) mRNA expression was quantitated with qRT–PCR (forward primer 5’ AAGATTCCAGCGCCCATTCA 3’ and reverse primer 5’ TGAGGAACGAACACGACTGG 3’), ISL2 (GenBank accession no. NM_145805) mRNA expression was quantitated with qRT–PCR (forward primer 5’ CTGCAAGCGGGACTACGTC 3’ and reverse primer 5’ CACTCGATGTGGTACACGC 3’), CD31 (GenBank accession no. AF281301) mRNA expression was quantitated with qRT–PCR (forward primer 5’ TTGAGACCAGCCTGATGAAACCCT 3’ and reverse primer 5’ TCCGTTTCCTGGGTTCAAGCGATA 3’), VEGFA (GenBank accession no. NM_001171623) mRNA expression was quantitated with qRT–PCR (forward primer 5’ AAGGAGGAGGGCAGAATCAT 3’ and reverse primer 5’ ATCTGCATGGTGATGTTGGA 3’), Vimentin (GenBank accession no. NM_003380.5) mRNA expression was quantitated with qRT–PCR (forward primer 5’ ACGTCTTGACCTTGAACGCA 3’ and reverse primer 5’ TCTTGGCAGCCACACTTTCA 3’) and normalized to GAPDH (GenBank accession no. JN613429.1) (forward primer 5’ CCTGGAGAAACCTGCCAAGT 3’ and reverse primer 5’ GCCAAATTCATTGTCGTACCA 3’) through standard ΔΔCt method as we described previously (2).

## Statistical analysis

Raw RNA sequencing counts and the corresponding clinical information of the glioma patients were obtained from a dataset in The Cancer Genome Atlas (TCGA, https://portal.gdc.cancer.gov/). Data are shown as the means ± standard deviations of the means. P < 0.05 was considered to indicate of a statistically significant difference. Kaplan–Meier survival analysis with the log-rank test was also used to compare the difference in survival between the ISL2^High^ and ISL2^Low^ groups. For Kaplan–Meier curves, p values and hazard ratios with 95% confidence intervals were calculated by the log-rank test and univariate Cox proportional hazards regression analysis, respectively. All analytical methods above and R packages were implemented using R software version v4.0.3 (The R Foundation for Statistical Computing, 2020). p < 0.05 was considered statistically significant.

## References

1. L. Qi, Z. Y. Wang, X. R. Shao, M. Li, S. N. Chen, X. Q. Liu, S. Yan, B. Zhang, X. D. Zhang, X. Li, W. Zhao, J. A. Pan, B. Zhao and X. D. Zhang: ISL2 modulates angiogenesis through transcriptional regulation of ANGPT2 to promote cell proliferation and malignant transformation in oligodendroglioma. *Oncogene*, 39(37), 5964-5978 (2020) doi:10.1038/s41388-020-01411-y

2. Y. Huang, L. Qi, M. Kogiso, Y. Du, F. K. Braun, H. Zhang, L. F. Huang, S. Xiao, W. Y. Teo, H. Lindsay, S. Zhao, P. Baxter, J. M. F. Su, A. Adesina, J. Yang, S. Brabetz, M. Kool, S. M. Pfister, M. Chintagumpala, L. Perlaky, Z. Wang, Y. Zhou, T. K. Man and X. N. Li: Spatial Dissection of Invasive Front from Tumor Mass Enables Discovery of Novel microRNA Drivers of Glioblastoma Invasion. *Adv Sci (Weinh)*, e2101923 (2021) doi:10.1002/advs.202101923
